# Ion antiport accelerates photosynthetic acclimation in fluctuating light environments

**DOI:** 10.1038/ncomms6439

**Published:** 2014-11-13

**Authors:** Ute Armbruster, L. Ruby Carrillo, Kees Venema, Lazar Pavlovic, Elisabeth Schmidtmann, Ari Kornfeld, Peter Jahns, Joseph A. Berry, David M. Kramer, Martin C. Jonikas

**Affiliations:** 1 Department of Plant Biology, Carnegie Institution for Science, 260 Panama Street, Stanford, California 94305, USA; 2 Plant Research Laboratory, Michigan State University, R106 Plant Biology Building, East Lansing, Michigan 48824-1312, USA; 3 Dpto de Bioquímica, Biología Celular y Molecular de Plantas, Estación Experimental del Zaidín, Consejo Superior de Investigaciones Científicas, c/. Profesor Albareda 1, 18008 Granada, Spain; 4 Biochemie der Pflanzen, Heinrich-Heine-Universität Düsseldorf, Universitätsstraße 1, 40225 Düsseldorf, Germany; 5 Department of Global Ecology, Carnegie Institution for Science, 260 Panama Street, Stanford, California 94305, USA

## Abstract

Many photosynthetic organisms globally, including crops, forests and algae, must grow in environments where the availability of light energy fluctuates dramatically. How photosynthesis maintains high efficiency despite such fluctuations in its energy source remains poorly understood. Here we show that *Arabidopsis thaliana* K^+^ efflux antiporter (KEA3) is critical for high photosynthetic efficiency under fluctuating light. On a shift from dark to low light, or high to low light, *kea3* mutants show prolonged dissipation of absorbed light energy as heat. KEA3 localizes to the thylakoid membrane, and allows proton efflux from the thylakoid lumen by proton/potassium antiport. KEA3’s activity accelerates the downregulation of pH-dependent energy dissipation after transitions to low light, leading to faster recovery of high photosystem II quantum efficiency and increased CO_2_ assimilation. Our results reveal a mechanism that increases the efficiency of photosynthesis under fluctuating light.

## Abstract (web summary)

Plants must respond rapidly to unpredictable variations in light intensity to maximize photosynthetic efficiency. Here Armbruster *et al.* identify a potassium antiporter that is critical for accelerating proton fluxes across thylakoid membranes and minimizing energy loss in fluctuating light conditions.

## Graphical abstract

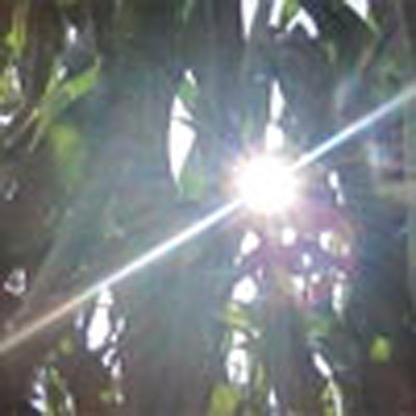

Efficient conversion of energy is a critical challenge for all organisms. Energy in the environment is available either as chemical potential or light. Uniquely, the energy available as light can undergo extreme fluctuations in amplitude on short time scales. Photon flux can change 100-fold within seconds, for example, due to shade from clouds or leaves moving in the wind^1^,^2^. Understanding the mechanisms that underlie efficient photosynthesis in fluctuating light can open doors to increased crop yields, and more broadly can reveal fundamental principles of robust energy conversion systems.

The light reactions of photosynthesis produce NADPH and ATP to power carbon fixation. Light-harvesting antennae absorb photons and transfer their energy to the photosystems, which pump electrons from water to NADPH. This process is coupled to proton translocation across the thylakoid membrane into the thylakoid lumen, which drives ATP synthesis^3^,^4^. Under high light, the high proton concentration in the thylakoid lumen activates energy-dependent quenching (qE), a mechanism that dissipates absorbed light energy in the PSII antenna as heat^5^. qE is thus part of a negative feedback loop that decreases energy transfer to PSII by up to 75% in response to excess light^6^,^7^,^8^.

To maximize yields under continuously changing light intensities, the PSII antenna must rapidly alternate between dissipation of excess absorbed energy under high light, and efficient harvesting of photons for photosynthesis under limiting light^9^. On transition from high to limiting light, the qE energy dissipation mechanism is downregulated to increase the energy available to PSII. However, this downregulation occurs on a timescale of minutes, producing an extended period where heat dissipation of light energy starves the photosystems of excitation energy. Simulations predict that the slow downregulation of heat dissipation mechanisms can reduce the photosynthetic efficiency by ~10% in crop canopies^10^. The design principles that determine the kinetics of qE downregulation remain unknown^11^, motivating a search for molecular factors that affect its dynamics.

Here we show that ion antiport activity across the thylakoid membrane accelerates photosynthetic acclimation after a change in light intensity from dark to low light, or from high to low light. In the model plant *Arabidopsis thaliana* (hereafter ‘*Arabidopsis*’), K^+^ efflux antiporter 3 (KEA3) is localized in the thylakoid membrane and mediates H^+^/K^+^ antiport. After each transition to low light, KEA3 allows proton efflux from the lumen, which accelerates the downregulation of pH-dependent energy quenching, and transiently increases photosystem II quantum efficiency and CO_2_ fixation. Our findings demonstrate that a K^+^/H^+^ antiport activity mediated by KEA3 increases photosynthetic efficiency under fluctuating light conditions.

## Results

### KEA3 is co-regulated with photosynthesis-related genes

The previously established regulation of qE by thylakoid pH^5^ led us to hypothesize that proton/cation antiporters in the thylakoid membrane could regulate the dynamics of qE. We therefore searched for proton/cation antiporters in *Arabidopsis* that were transcriptionally co-regulated with photosynthesis-related genes. The expression of *KEA3* was strongly correlated with genes annotated with the gene ontology (GO) term ‘photosynthesis’ (Supplementary Fig. 1a, *P*<10^−12^, Mann–Whitney *U*-test). Conversely, the most significantly enriched GO term among the 100 genes whose expression was most highly correlated with *KEA3* was ‘photosynthesis’ (*P*<10^−41^). KEA3 is highly conserved among photosynthetic eukaryotes (Supplementary Fig. 1b).

### KEA3 is localized in the thylakoid membrane

**Figure 1:**
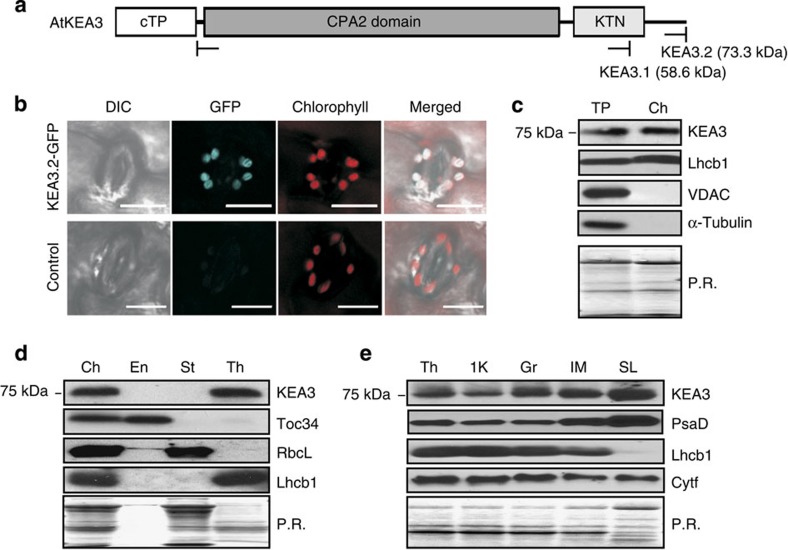
KEA3 localizes to the thylakoid stromal lamellae. (**a**) KEA3.2 has an amino-terminal chloroplast-targeting peptide, a cation/proton exchanger (CPA2) domain and a putative KTN domain. (**b**) Differential interference contrast, GFP and chlorophyll fluorescence were imaged in leaf guard cells from *Arabidopsis* WT plants and *kea3-1* plants transformed with *KEA3.2-GFP*. Scale bar, 10 μm. (**c**–**e**) KEA3 abundance was measured by western blot in: (**c**) Total protein (TP) and chloroplast (Ch) protein extracts; (**d**) Ch subfractionated into envelope (En), stroma (St) and thylakoids (Th); (**e**) Th separated into the fraction pelleted by 1,000*g* (1 K), grana (Gr), intermediate membranes (IM) and stromal lamellae (SL). (**c**–**e**) Ponceau red (P.R.) stains of membranes after protein transfer before immunodetection are shown as loading controls.

Two splice forms of *KEA3* are annotated in *Arabidopsis*: *KEA3.1* and *KEA3.2*. Both encode a predicted chloroplast transit peptide and a cation/H^+^ antiporter (CPA2) domain (Fig. 1a, Supplementary Fig. 2a). *KEA3.2* additionally encodes a carboxyl-terminal (C-terminal) K^+^ transport/nucleotide-binding (KTN) domain, which is truncated in *KEA3.1*. Size analyses of the endogenous KEA3 protein and tagged versions of both isoforms indicate that KEA3.2 is the major protein isoform in leaves (Supplementary Fig. 2). Recently, C-terminal fluorescent protein fusions of KEA3.1 and KEA3.2 were localized to the chloroplast and the Golgi, respectively^12^,^13^. In contrast to previous work, we observed KEA3.2-green fluorescent protein (GFP) in the chloroplast (Fig. 1b). We confirmed the localization of the endogenous KEA3 to the chloroplast in wild-type (WT) leaves by western blot of total and chloroplast protein extracts (Fig. 1c, Supplementary Fig. 3a). Subfractionation of WT chloroplasts demonstrated that endogenous KEA3 is located in the thylakoid membrane and is enriched in the stromal lamellae (Fig. 1d,e, Supplementary Fig. 3b,c).

### KEA3 accelerates photosynthetic acclimation to low light

**Figure 2:**
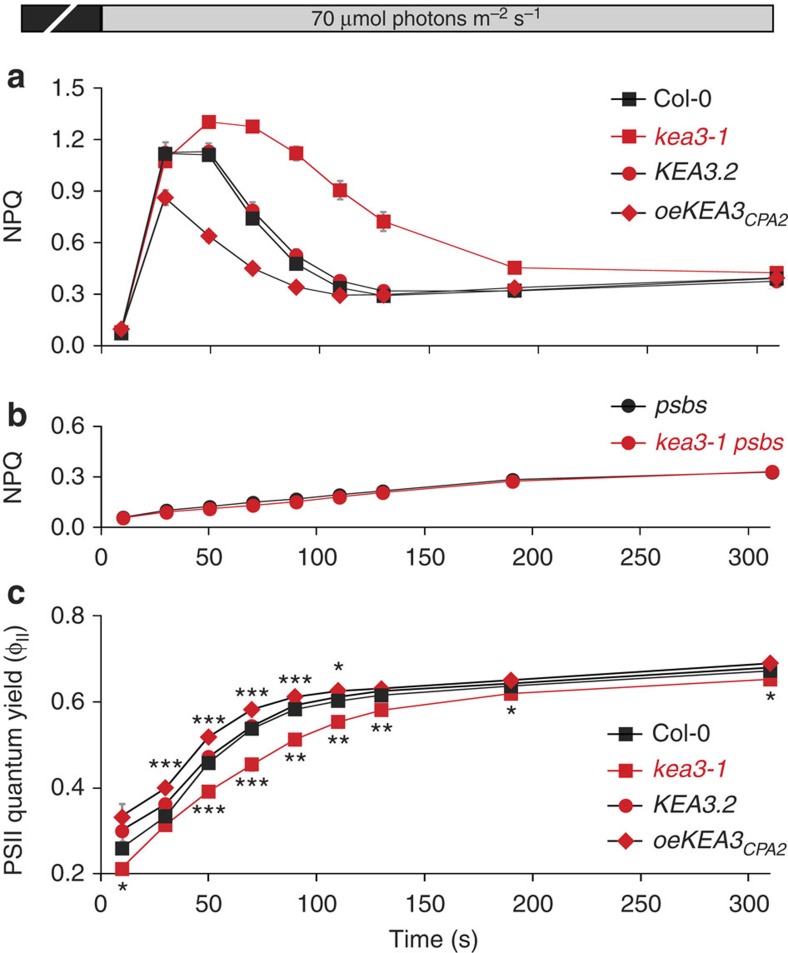
KEA3 accelerates qE relaxation on transition from dark to low light. (**a**) NPQ induction in detached leaves of 3-week-old Col-0, *kea3-1* and *kea3-1* expressing *KEA3.2-GFP* (*KEA3.2*) or overexpressing *KEA3*_*CPA2*_*-GFP* (*oeKEA3*_*CPA2*_) was measured at 70 μmol photons m^−2^ s^−1^ after 30 min dark incubation. (**b**) The extra NPQ in *kea3* mutants requires PsbS. NPQ induction in detached leaves of 2-week-old *psbs* and *kea3-1 psbs* mutant plants was measured as in **a**. (**c**) The higher transient NPQ in *kea3* decreases the PSII quantum yield (Φ_II_), and *oeKEA3*_*CPA2*_ show transiently increased Φ_II_. Φ_II_ was calculated from the same experiment as **a**. Asterisks indicate time points where *kea3-1* or *oeKEA3*_*CPA2*_ differ significantly from WT (*0.01<*P*<0.04, **0.001<*P*<0.01, ***P<0.001, Student’s *t*-test). (**a**–**c**) Error bars represent s.e.m. (*n*=6).

We analysed the phenotypes of two *kea3* mutant alleles, *kea3-1* and *kea3-2* (Supplementary Fig. 2). These mutants displayed no measurable defects in growth, leaf colouration or protein composition of the photosynthetic apparatus (Supplementary Fig. 4). To characterize potential defects in regulation of photosynthesis, we performed chlorophyll fluorescence measurements across various light conditions. We observed a striking perturbation of the kinetics of non-photochemical energy quenching (NPQ) on a shift from dark to low-light conditions. In flowering plants, a shift from a dark-acclimated state to light-limiting conditions causes a transient rise in NPQ, which is attributed to qE^14^. The *kea3* mutants displayed a higher maximum NPQ and a delay in its peak and relaxation (Fig. 2a, Supplementary Fig. 5a). This *kea3* phenotype was complemented by expressing a KEA3.2-GFP fusion protein that accumulated at levels similar to the native protein (Fig. 2a, Supplementary Fig. 6). Remarkably, plants overexpressing the amino-terminal part of KEA3 containing the chloroplast transit peptide and CPA2 domains (*oeKEA3*_*CPA2*_) showed accelerated qE relaxation kinetics. When shifted from dark to higher light intensities, *kea3* mutants displayed WT-like induction kinetics of NPQ (Supplementary Fig. 5c,e).

The transient nature of the additional NPQ observed on a shift to low light suggested that qE was perturbed in *kea3*. qE is known to be modulated by the luminal pH and zeaxanthin, and normal qE kinetics require the protein PsbS^14^. *kea3psbs* double mutants exhibited the same NPQ kinetics as *psbs* single mutants (Fig. 2b, Supplementary Fig. 7a), indicating that PsbS is required for the additional NPQ observed in *kea3*. Double mutants of *kea3* with *npq1* (lacking zeaxanthin) and *npq2* (overaccumulating zeaxanthin) revealed that the NPQ phenotype was modulated by levels of zeaxanthin (Supplementary Fig. 7b,c). Application of high concentrations of nigericin, which inhibit qE^15^, to leaves, abolished the rapid component of NPQ in both WT and *kea3* (Supplementary Fig. 8a). Thus, the additional NPQ observed in *kea3* shows the key characteristics of qE.

The transient increase in NPQ was accompanied by lower PSII quantum yield (Φ_II_) in *kea3* (*P*<0.004, Student's *t*-test, Fig. 2c), indicating decreased linear electron flow through PSII. This decrease in Φ_II_ was PsbS dependent (Supplementary Fig. 7e), suggesting that it was caused by an increase in qE. In *oeKEA3*_*CPA2*_, Φ_II_ was transiently higher than in WT (Fig. 2c). We conclude that KEA3 accelerates qE relaxation, allowing more absorbed light energy to be used to drive electron flow through PSII.

### KEA3 likely mediates K^+^/H^+^ antiport

**Figure 3:**
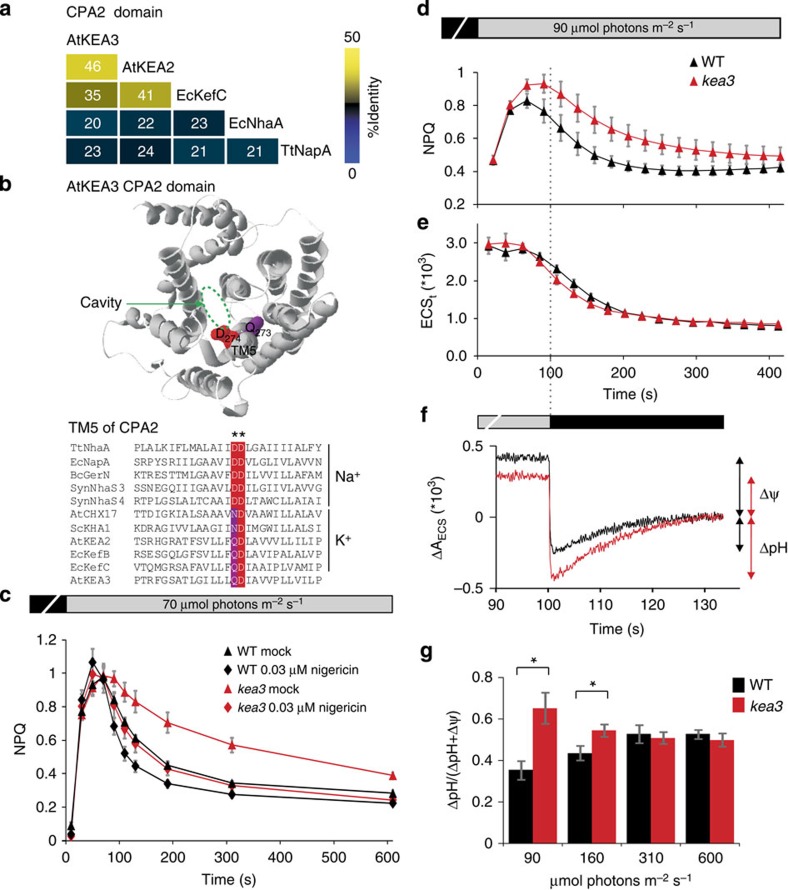
KEA3 regulates the composition of the p.m.f. by mediating potassium/proton antiport. (**a**) The CPA2 domain of KEA3 is homologous to that of known K^+^/H^+^ antiporters. A percent identity matrix is shown for *Arabidopsis* KEA2 and KEA3, *E. coli* KefC and NhaA and *T. thermophilus* NapA. (**b**) The locations of two key substrate-binding amino acid residues in TM5 of the CPA2 domain^19^ are shown on a model of KEA3. (**c**) Low levels of Nigericin complement the *kea3* NPQ phenotype. NPQ induction on transition from dark to 70 μmol m^−2^ s^−1^ light was measured in WT (Ws) and *kea3-2* leaves incubated in water (mock) or 0.03 μM nigericin. (**d**,**e**) p.m.f. kinetics in *kea3* mutants are largely unaffected. NPQ and ECS_t_ (which reports the magnitude of the p.m.f.) were measured near-simultaneously in WT (Ws) and *kea3-2* by Chl fluorescence and dark-induced relaxation kinetics, respectively, in single leaves during a transition from dark to 90 μmol m^−2^ s^−1^. (**f**) After 100 s of low light, *kea3* mutants show increased ΔpH and decreased Δψ. Full ECS decay kinetics were recorded after 100 s of low light (90 μmol m^−2^ s^−1^) to measure ΔpH and Δψ in WT (Col-0) and *kea3-1*. The average of six independent measurements per genotype was plotted as a moving average with interval 5 (see also Supplementary Fig. 9). (**g**) The experiment in **f** was repeated at different light intensities, and the fraction of the p.m.f. contributed by ΔpH was plotted. Asterisks indicate where WT and *kea3* differ significantly (*P<0.04, Student’s *t*-test). (**c**–**e**,**g**) Error bars represent s.e.m. (*n*=6).

To gain insights into the identity of the cations transported by the CPA2 domain of KEA3, we used phylogenetics. KEA3 shows more sequence homology to experimentally validated K^+^/H^+^ antiporters AtKEA2 (ref. 16) and EcKefC^17^ than to Na^+^/H^+^ antiporters EcNhaA and TtNapA (Fig. 3a). Comparison of two amino acid residues critical for Na^+^/H^+^ binding in Na^+^/H^+^ antiporters of the CPA2 family^18^,^19^ indicates that presence of an Aspartate–Aspartate motif strictly correlates with Na^+^/H^+^ antiport activity, while conversion of the first residue to Asparagine or Glutamine correlates with K^+^/H^+^ antiport activity^16^,^17^,^20^ (Fig. 3b). Like AtKEA2 and EcKefC, KEA3 has a Glutamine–Aspartate motif. Finally, the presence of the K^+^ transport-related KTN domain in KEA3 further supports a K^+^/H^+^ antiporter activity^21^,^22^.

We reasoned that if KEA3 is a K^+^/H^+^ antiporter, low concentrations of the small molecule electroneutral K^+^/H^+^ antiporter nigericin might rescue the *kea3* phenotype. Strikingly, infiltration of leaves with 0.03 μM nigericin fully rescued the qE phenotype (Fig. 3c, Supplementary Fig. 8a). This result strongly suggests that KEA3 accelerates the qE relaxation by electroneutral K^+^/H^+^ antiport.

### KEA3 decreases the ΔpH component of the proton motive force

If KEA3 is a K^+^/H^+^ antiporter, it could alter the proton concentration (ΔpH) and/or electric potential (Δψ) components of the proton motive force (p.m.f.). We performed near-simultaneous measurements of NPQ as a readout of ΔpH, and of the magnitude of the carotenoid electrochromic shift (ECS_t_)^23^ as a readout of total p.m.f. (ΔpH+Δψ), during the transition from dark to low light (Fig. 3d,e). The dynamics of the p.m.f. were largely unchanged in *kea3*, but the ratio of NPQ to ECS_t_ was higher than in WT, suggesting that KEA3 transiently decreases the contribution of ΔpH to the proton motive force.

We measured both ΔpH and Δψ after 100 s illumination by turning off the light and observing the extended kinetics of the ECS^24^. These measurements indicated that after 100 s of low light, the ΔpH is greater in *kea3* than in WT (*P*<0.04, Student's *t*-test, Fig. 3f,g, Supplementary Fig. 8b), consistent with the observed increased NPQ in *kea3* at this time point (Figs 2a and 3d, Supplementary Fig. 5a). This difference was not present at higher light intensities. These data further support the idea that KEA3 decreases the contribution of ΔpH and increases the contribution of Δψ to p.m.f.

### KEA3 accelerates acclimation from high to low light

**Figure 4:**
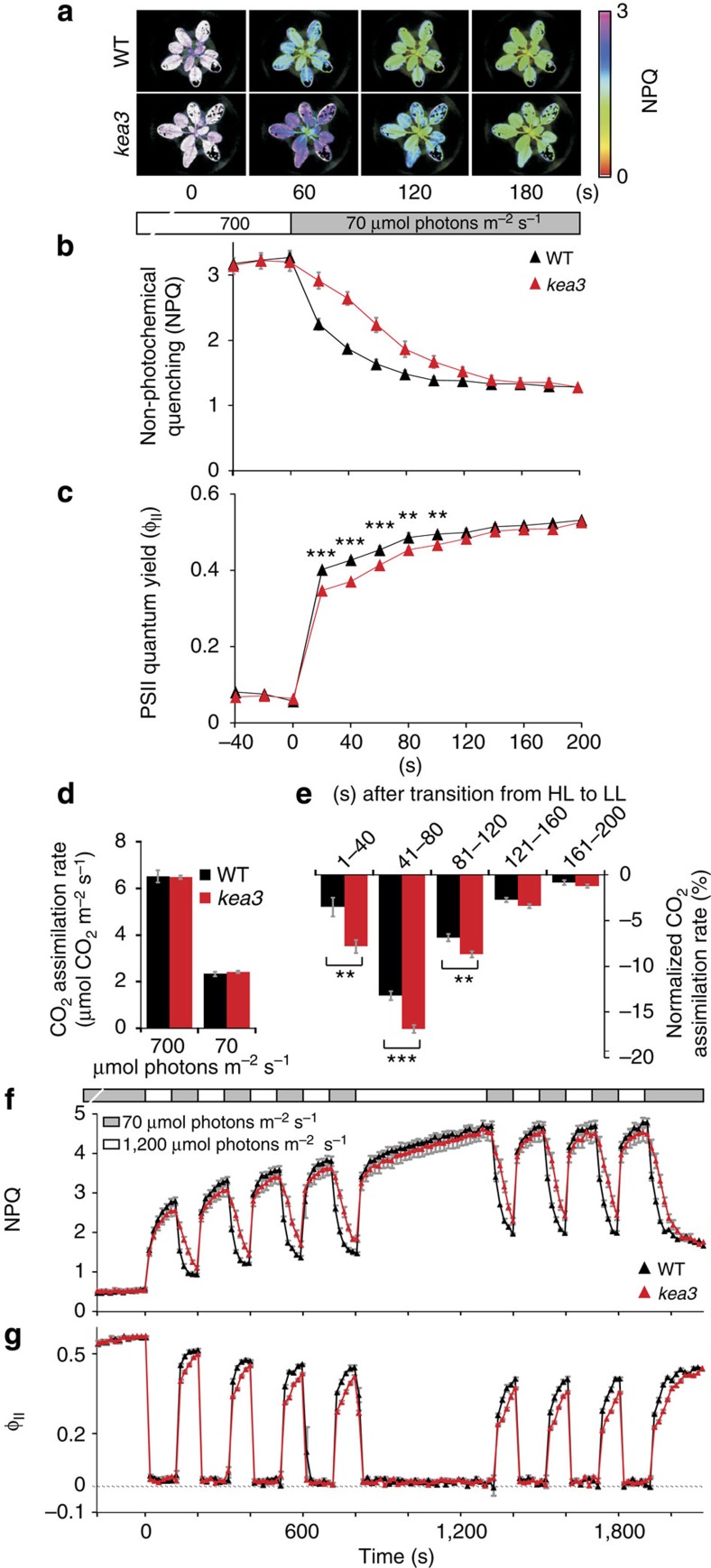
KEA3 regulates the dynamics of photosynthesis during transitions from high to low light. (**a**,**b**) NPQ relaxation on transition from high to low light is delayed in *kea3* (*kea3-2*) as compared with WT (Ws). (**c**) Φ_II_ is decreased during transition from high to low light. Φ_II_ was calculated from the same measurement as in **a**,**b**. (**d**) WT (Ws) and *kea3* (*kea3-2*) show similar CO_2_ assimilation rates during steady-state high (HL, 700 μmol photons m^−2^ s^−1^) and low light (LL, 70 μmol photons m^−2^ s^−1^). (**e**) The normalized CO_2_ assimilation rate (shown as a percentage of the difference between HL and LL) is decreased in *kea3* (*kea3-2*). (**f**) In fluctuating light, NPQ relaxation is delayed after each transition from high to low light in *kea3-2*. (**g**) Φ_II_ is decreased in *kea3-2* during each transition from high to low light. Φ_II_ was calculated from the same measurement as in (**f**). (**c**,**e**) Asterisks represent significantly lower values in *kea3-2* (**0.001<*P*<0.01, ***P<0.001, Student’s *t*-test). (**b**–**g**) Error bars represent s.e.m. (**b**–**e**: *n*=7; **f**,**g**: *n*=4).

Transitions from high to low light are common in many growth environments, including crop canopies^1^. We therefore asked whether KEA3 influences the dynamics of photosynthesis during transitions from high to low light. After a sudden shift from high to low light, NPQ in WT *Arabidopsis* plants decayed over ~80 s to a new, lower level (Fig. 4a,b, Supplementary Fig. 9a). Strikingly, NPQ decayed more slowly in *kea3*, while high and low light NPQ values were comparable to WT. The half time of the NPQ decay in *kea3* was twice as long as that in WT (Col-0: 24.8±1.4 s, *kea3-1*: 47.6±3.0 s, *P*=4.1 × 10^−5^; Ws: 25.9±1.4 s, *kea3-2*: 51.8±2.0 s *P*=1.8 × 10^−6^ (± s.e.m., Student’s *t*-test)). These data suggest that KEA3 plays a key role in accelerating the decay of NPQ during transitions from excess to limiting light conditions.

We observed decreased Φ_II_ in *kea3* (*P*<0.02, Student’s *t-*test) over a similar time interval to the NPQ defect (Fig. 4c, Supplementary Fig. 9b). As during transition from dark to low light (Fig. 2a,c), these data suggest that the slower relaxation of NPQ in *kea3* results in a transient decrease in linear electron flow relative to WT.

We measured CO_2_ assimilation during the same transition. While CO_2_ assimilation rates of *kea3* and WT plants were similar under steady-state high and low light, *kea3* exhibited a period of lower CO_2_ assimilation than WT immediately after a transition from high to low light (Fig. 4d,e, *P*<0.007, Supplementary Fig. 9c,d, *P*<0.04). We conclude that following a high- to low-light transition, KEA3 accelerates the downregulation of qE, thus allowing a larger fraction of the absorbed light energy to drive linear electron flow and CO_2_ fixation.

### KEA3 improves photosynthetic efficiency in fluctuating light

To investigate the role of KEA3 under continuously fluctuating light, we exposed plants to alternating cycles of 140 s at high light and 100 s at low light. *kea3* mutants consistently showed slower NPQ relaxation and Φ_II_ recovery than WT after each transition (Fig. 4f,g and Supplementary Fig. 10). These phenotypes were present regardless of whether the fluctuations were preceded by 10 min of high light or 10 min of low light, suggesting that KEA3’s function is not extensively affected by preceding light conditions. NPQ and Φ_II_ in *kea3* reached WT levels after the fluctuations stopped, further supporting the role of KEA3 specifically in light transitions. We conclude that KEA3 improves photosynthetic efficiency under continuously fluctuating light.

## Discussion

Our data are consistent with the following intriguing model: (i) The dynamics of qE relaxation are dictated by the dynamics of the thylakoid luminal pH. (ii) After a sudden transition to low light, the ATP synthase alone cannot sustain sufficient proton efflux to enable rapid downregulation of qE. (iii) KEA3 overcomes this limitation by providing an additional route for proton efflux, thereby accelerating qE relaxation and increasing photosynthetic yield. KEA3’s electroneutral activity would provide two advantages for proton efflux over a simple proton channel: (i) it retains the Δψ component of the p.m.f. and (ii) it can rapidly mediate a large flux because its activity does not generate an opposing electric field.

We note that the combined activities of KEA3 and the recently identified thylakoid potassium channel TPK3 (ref. 25) would dissipate the p.m.f. We thus anticipate that under most conditions, the activities of these two proteins are tightly regulated to minimize waste of energy. However, under some conditions causing harmful excess ΔpH^26^, KEA3 and TPK3 together may form a safety valve to allow dissipation of p.m.f.

Intriguingly, the KTN domain of KEA3 could regulate KEA3 activity in response to the redox state of the stroma. In *E. coli*, NADH binds to the KefC KTN domain and represses KefC activity^17^. The observation that the *kea3* phenotype is specific to transitions to low light suggests that KEA3 might similarly be repressed by NADPH (or alternatively, activated by NADP). KEA3 could thus be a key component of a feedback loop, which detects an oxidized stroma and facilitates reduction of oxidized PSI electron acceptors by downregulating qE.

Our work demonstrates that the kinetics of qE decay influence photosynthetic efficiency. Our discovery that plants have a built-in machinery to accelerate these kinetics makes KEA3 a new target for efforts to improve crop productivity. Excitingly, overexpression of the KEA3 CPA2 domain produces a transient increase in the relative CO_2_ assimilation rate after transition from high to low light (Supplementary Fig. 9). However, the overexpression of the CPA2 domain appears to also result in undesirable photodamage in high light. It is possible that improved regulation of KEA3 would overcome this limitation. Thus, the future investigation of KEA3 should yield both fascinating insights into the core logic of photosynthesis regulation, as well as keys to possible enhancements in crop yields.

## Methods

### Plant material, propagation and growth conditions

The *kea3-1* mutant (Gabi_170G09) in the Col-0 genetic background originates from the GABI-KAT collection^27^. The *kea3-2* mutant (FLAG_493_C01) in the Wassilewskija (Ws) genetic background originates from the FLAGdb/FST T-DNA collection^28^. The *aba1* mutant (Salk_059469) in the Col-0 genetic background originates from the SALK T-DNA collection^29^. The *npq1* and *psbs* mutants have been described previously^7^,^8^.

Plants expressing *KEA3.1* and *KEA3.2* with a C-terminal HA tag were generated by introducing the *KEA3* coding regions (for primers, see Supplementary Table 1) into the Gateway plant expression vector pJCV55 (ref. 30) downstream of a Cauliflower mosaic virus 35S promoter. Constructs for expressing KEA3.02 and KEA3_CPA2_ with a C-terminal eGFP tag were generated in a similar manner, but with vector pB7FWG2. Flowers of *kea3-1* mutant plants were transformed with the *KEA3* constructs by floral dip^31^. The plants were then transferred to the greenhouse and seeds were collected after 3 weeks. Individual transgenic plants were selected on the basis of their resistance to kanamycin (pJCV55) or Basta (pB7FWG2) on sterile 1% agar media containing 1 × MS nutrients (PhytoTechnology Laboratories) and 0.05 g l^−01^ MES, adjusted to pH 5.7 with KOH. Before transfer to soil, Kanamycin- and Basta-resistant plants were additionally screened for red fluorescent protein and GFP expression, respectively, using an M165 FC fluorescence microscope (Leica) to verify expression of the transgene (the T-DNA deriving from pJCV55 carries an additional red fluorescent protein-encoding gene). The presence of the *KEA3* transgene in transformants was confirmed by PCR and its expression was confirmed by protein immunoblot analyses.

WT and mutant *Arabidopsis* plants were grown on soil under controlled conditions in the greenhouse (matted daylight supplemented with high pressure sodium 400 W lamps, ~100 μmol photons m^−2^ s^−1^ on leaf surfaces, 14/10 h light/dark cycle, constant temperature of 20 °C) or in growth chambers (10 h light, 22 °C/14 h dark, 23 °C, with a light intensity of 100 μmol photons m^−2^ s^−1^).

### Nucleic acid analysis

*Arabidopsis* DNA was extracted using RedExtract-N-Amp (Sigma). T-DNA insertion junction sites were recovered by PCR using combinations of insertion- and gene-specific primers (see Supplementary Table 1), and then sequenced. For reverse transcriptase-PCR (RT–PCR) analysis and cDNA synthesis, RNA was extracted from leaves using the RNeasy plant mini kit (Qiagen) according to the manufacturer’s instructions.

The cDNA for cloning purposes was synthesized using Superscript III (Invitrogen). RT–PCR was performed with SuperScript III One-Step RT–PCR System (Invitrogen) and gene-specific primers (see Supplementary Table 1), both according to the manufacturer’s instructions.

### Fluorescence measurements

Room temperature Chl *a* fluorescence of whole leaves of 2–3-week-old *Arabidopsis* plants was measured using an imaging chlorophyll fluorometer (Open FluorCam 800-O/1010; Photon Systems Instruments) or the Dual-PAM 100 (Walz). NPQ during dark-to-light transitions was determined using the FluorCam by acclimating plants for 30 min to dark, followed by 5–10 min at indicated light intensities of red light. To measure Fm and Fm′, white light pulses (4,000 μmol photons m^−2^ s^−1^, duration 800 ms) were applied. NPQ induction and relaxation during dark to 50, 220 and 660 μmol photons m^−2^ s^−1^ transitions were monitored using the Dual-PAM by applying red actinic light and white light pulses (4,000 μmol photons m^−2^ s^−1^, duration 800 ms) at 20 s intervals. NPQ was calculated as (Fm−Fm′)/Fm′ and Φ_II_ as (Fm−Fs)/Fm.

NPQ relaxation half times were calculated as follows. A steady-state low light average NPQ value was subtracted from the first five data points during the NPQ relaxation period. The binary logarithm of these values was taken, and a linear fitting was performed to obtain a slope *s*. *t*_1/2_ was calculated as *t*_1/2_ (NPQ)=1/−s.

For nigericin rescue experiments, plants were dark adapted for 30 min. Then, leaves were pressed against sand paper to facilitate nigericin uptake and incubated in indicated concentrations of nigericin for 3 h in the dark before measuring NPQ during dark-to-light transitions at 70 μmol photons m^−2^ s^−1^.

### ECS measurements

Near-simultaneous ECS and fluorescence measurements as well as p.m.f. partitioning analysis were performed on a custom made spectrophotometer/chlorophyll fluorometer chamber with non-focusing optics using three different wavelengths (505, 520 and 535 nm) for deconvoluted ECS measurements^23^,^32^. Leaves were dark adapted for 30 min before the actinic light of 90 μmol photons m^−2^ s^−1^ was turned on. Each dark interval relaxation kinetic measurement was made by turning off the actinic light for 200 ms. p.m.f. parsing traces were obtained by turning off the actinic light at 100 s. The ECS steady-state (Δψ) and ECS inverse (ΔpH) were extracted from these traces as described previously^24^,^33^.

### CO_2_ assimilation measurements

Plants were grown in 50 ml Falcon tubes with a pierced bottom filled with Sunshine Mix 4 potting mix, placed in a dish containing 1–3 cm of water, under 100 μmol m^−2^ s^−1^ fluorescent light. For measurement, the Falcon tubes were inserted using a foam gasket into a LI-COR 6400-17 Whole Plant Arabidoposis Chamber. The chamber was placed under a Walz MAXI IMAGING-PAM with blue actinic LEDs, which ran the light programs and was used to simultaneously record chlorophyll fluorescence data. CO_2_ assimilation was measured with a reference CO_2_ concentration of 400 μmol mol^−1^ and a 500 μmol s^−1^ flow rate, averaging and recording data over 2 s intervals. The steady-state assimilation rates under high and low light were obtained for each plant by averaging the assimilation rate over the intervals (−40 to 0 s) and (+560 to +600 s), respectively, and normalizing the assimilation rate by the rosette area measured from IMAGING-PAM images with ImageJ. The transient CO_2_ assimilation was calculated as follows. For each plant, the data was normalized so that steady-state high light assimilation rate was 1, and the steady-state low light assimilation rate was 0. The average normalized assimilation rate in each time interval was then calculated for each plant.

### Leaf pigment analysis

Pigments were analysed by reverse-phase high-performance liquid chromatography^34^. For pigment extraction, leaf discs were frozen in liquid nitrogen and disrupted with beads in microcentrifuge tubes in the presence of acetone. After a short centrifugation, pigment extracts were filtered through a membrane filter (pore size 0.2 μm) and either used directly for high-performance liquid chromatography analysis or stored for up to 2 days at −20 °C.

### Computational analysis

*Arabidopsis KEA3.1* and *KEA3.2* (*At4g04850.1* and*.2*) DNA and protein sequences were retrieved from TAIR (The Arabidopsis Information Resource; www.arabidopsis.org). Subcellular localization signals were analysed by SUBA3 ( http://suba.plantenergy.uwa.edu.au/), which combines predictions from up to 22 prediction programs^35^, and by ChloroP ( http://www.cbs.dtu.dk/services/ChloroP)^36^.

For sequence comparisons, homologues of the *Arabidopsis* KEA3 protein (AtKEA3, Uniprot: Q9M0Z3) were identified by BLAST. Percent identity matrices were calculated for the CPA2 (Pfam00999) domains of *E. coli* NhaA (EcNhaA, P13738), *T. thermophilus* NapA (TtNapA, Q72IM4), *E. coli* KefC (EcKefC, P03819), AtKEA3, *Arabidopsis* KEA2 (AtKEA2, Q65272) and homologous AtKEA3 sequences from *Oryza sativa* (O.s., Q2QM48), *Physcomitrella patens* (P.p., A9SSR0) and *Chlamydomonas reinhardtii* (C.r., A8ISE8) by using ClustalOmega ( http://www.ebi.ac.uk/Tools/msa/clustalo/). For the homologous KEA3 sequences, an identity matrix was also calculated for the KTN domain (Pfam02254). To generate the KEA3 three-dimensional model, the TtNapA protein was used as a template by the Phyre2 server (http://www.sbg.bio.ic.ac.uk/~phyre2/)^37^. Sequence comparison of TM5 of the CPA2 domain of AtKEA3, EcNhaA, EcKefB (P45522) EcKefC, TtNapA, *Bacillus cereus* GerN (BcGerN, Q9KI10), Synechocystis NhaS3 (Q55190) and NhaS4 (Q5N3F5), *Arabidopsis* AtCHX17 (Q9SUQ7), AtKEA2 and *Saccharomyces cerevisiae* KHA1 (P40309) were performed using ClustalOmega.

*KEA3* co-expression analysis was performed by determining Pearson correlation coefficients of all *Arabidopsis* genes with *KEA3* using the GeneCAT database ( http://genecat.mpg.de). All genes in the database whose identifiers contained ‘At’ were used in this analysis. The 100 genes with the highest correlation values were analysed for the enrichment of specific GO terms by using AmiGO ( amigo.geneontology.org/cgi-bin/amigo/term_enrichment,^38^).

### Protein isolation, PAGE and immunoblot analyses

Total protein was isolated from leaves using the protein extraction buffer and corresponding protocol from Agrisera. Thylakoid membranes were isolated from leaves at 4 °C by shredding leaf tissue in 0.1 M Tricine/KOH pH 7.9, 400 mM Sorbitol, protease inhibitor cocktail (Sigma) using a Waring blender and filtering the homogenate through two layers of miracloth. The flow-through was centrifuged at 1,000*g* for 5 min. The resulting pellet was resuspended and incubated in 20 mM Hepes/KOH pH 7.6, 10 mM EDTA for 30 min to break remaining intact chloroplasts. Thylakoid membranes were then extracted by a centrifugation at 10,000*g* for 10 min. For SDS-polyacrylamide gel electrophoresis, proteins were fractionated on Tris-glycine gels (Bio-Rad). For Blue Native polyacrylamide gel electrophoresis, thylakoid membranes were solubilized with 0.7% β-*n*-dodecyl-D-maltoside (w/v) and separated by Blue Native gels (Invitrogen)^39^. For protein blot analysis, proteins were transferred to a nitrocellulose membrane, blocked with 5% (w/v) nonfat dry milk and hybridized with the antibodies indicated in the figure legends and text. Antibodies (except for α-KEA3, α-GFP and α-HA) were purchased from Agrisera. All antibodies were diluted in 50 mM Tris, 150 mM NaCl, 0.1% (v/v) Tween 20 and 5% (w/v) nonfat dry milk before use (α-Tubulin (AS10680) 1:3,000, VDAC (AS07212) 1:10,000, Lhcb1 (AS01004) 1:10,000, RbcL (AS03037) 1:20,000, TOC34 (AS07238) 1:2,000, PsaD (AS09461) 1:5,000, Cytf (AS08306) 1:10,000, CP47 (AS04038) 1:10,000 and PsbS (AS03032) 1:5,000. α-GFP (Invitrogen, A-6455) was diluted 1:2,000 and α-HA (Sigma, H6908) 1:2,500. Uncropped scans of the western blots are shown in Supplementary Fig. 3. AtpB and PsbS signals were quantified by densitometric analysis of western bands using NIH ImageJ software and associated plug-ins ( http://imagej.nih.gov/ij/).

For immunolocalization of KEA3, an antibody against the specific peptide sequence NQLGRKAADFLDERLDPGE (present in both KEA3.1 and KEA3.2 isoforms) was generated in rabbits by Yenzym and a dilution of 1:100 in 50 mM Tris, 150 mM NaCl, 0.01% (v/v) and 5% (w/v) nonfat dry milk was used for hybridization.

### Determination of KEA3 localization

Leaves of Col-0 or *kea3-1* plants expressing *KEA3.2-GFP* were analysed using a Leica SP5 AOBS Point Scanning Confocal Microscope. GFP and chlorophyll autofluorescence (Chl) were excited at 488 nm and signals were collected at 660–736 nm (Chl) and 495–515 nm (GFP).

*Arabidopsis* chloroplasts were isolated by a two-step Percoll gradient centrifugation^40^. The homogenized leaf tissue was applied to a two-step Percoll gradient and intact chloroplasts were collected from the interphase. For chloroplast fractionation, chloroplasts were hypotonically lysed in 10 mM Tris/HCl, pH 8.0 and 1 mM EDTA, pH 8.0, at a chlorophyll concentration of 2 mg ml^−1^, and loaded onto a three-step Sucrose gradient^41^. After centrifugation (30,000*g*; 4 °C, 1 h), the upper phase (containing the stroma), the upper interphase (with the envelope membranes) and the pellet (thylakoids) were collected and the proteins were precipitated^42^.

Thylakoid fractionation was performed according to Kyle *et al.*^43^ with minor modifications. Thylakoid membranes at 0.4 mg chlorophyll ml^−1^ in 15 mM Tricin/ KOH pH7.8, 0.1 M Sorbitol, 15 mM NaCl, 5 mM MgCl_2_ were treated with 0.15% digitonin (w/v) for 1 min and then diluted 10-fold with the same buffer. Samples were then centrifuged at 1,000; 10,000; 40,000; and 150,000*g* to yield the different fractions.

## Author contributions

U.A. and M.C.J. conceived the study; U.A., P.J., K.V., D.M.K. and M.C.J. designed the experiments; U.A. performed KEA3 localization, generation and analysis of double mutants; U.A. and E.S were responsible for the generation of *KEA3-GFP* expressing plants; U.A. performed the analysis; U.A. and L.P. performed Chl *a* fluorescence measurements; L.R.C. performed simultaneous Chl *a* fluorescence and ECS measurements, parsing experiments; U.A., M.C.J., A.K. and J.A.B. performed simultaneous Chl *a* fluorescence and gas exchange measurements; L.P. performed the pigment analysis; U.A. and K.V. performed the phylogenetic analyses; U.A. and M.C.J. wrote the paper.

## Additional information

**How to cite this article:** Armbruster, U. *et al.* Ion antiport accelerates photosynthetic acclimation in fluctuating light environments. *Nat. Commun.* 5:5439 doi: 10.1038/ncomms6439 (2014).

## Supplementary Material

Supplementary Information Supplementary Figures 1-10, Supplementary Table 1.
