# Supplementary material for: Ion antiport accelerates photosynthetic acclimation in fluctuating light environments
Source: Nat Commun. 2014 Nov 13;5:5439. doi: 10.1038/ncomms6439 (PMC4243252; doi:10.1038/ncomms6439)
Supplement: Supplementary Information — Supplementary Figures 1-10, Supplementary Table 1. [file ncomms6439-s1.pdf]

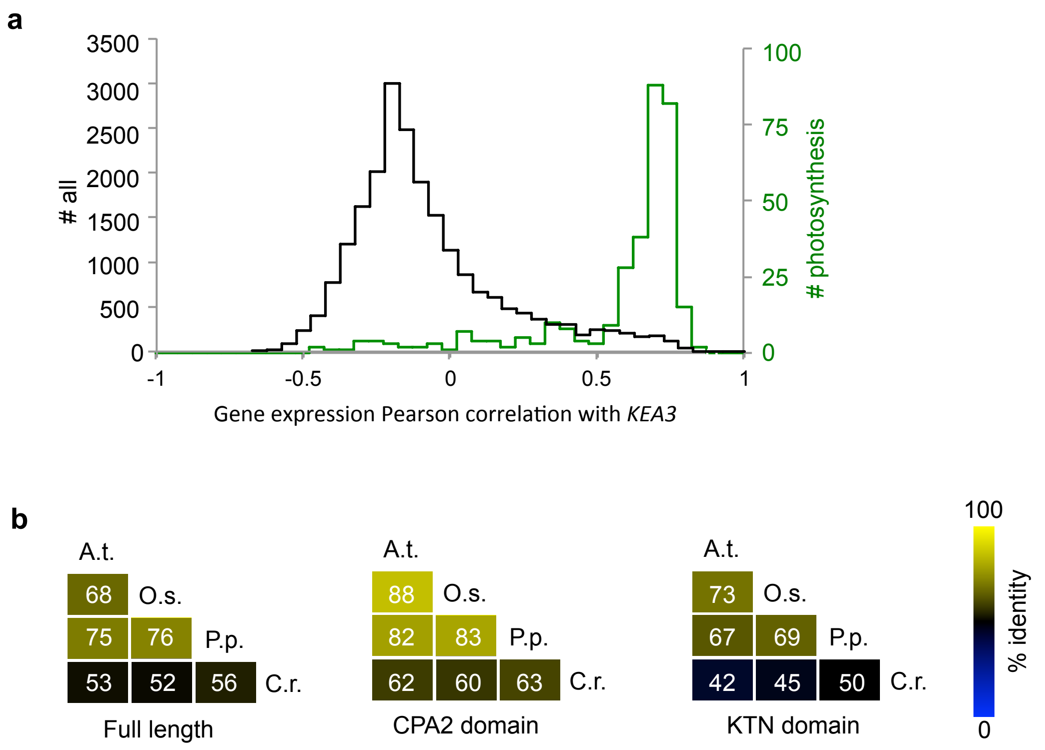

Supplementary Figure 1 | *KEA3* is transcriptionally co-regulated with genes involved in photosynthesis and is highly conserved in photosynthetic eukaryotes. a, We looked for enrichment of GO terms among the 100 genes whose expression was most highly correlated with *KEA3*. The most highly enriched GO terms were "photosynthesis" (GO:0015979; p=10^{-41}), “phosphate containing compound metabolic process” (GO:0006796, p=10^{-38}; this set includes most photosynthesis genes), "pentose phosphate shunt" (GO:0006098) and "NADPH regeneration" (GO:0006740, both p= 10^{-38}). Pearson correlation coefficients of *KEA3* with all genes (black) and genes belonging to the GO category “photosynthesis” (green) are plotted. **b,** The conservation of KEA3 proteins and its CPA2 and KTN domains was analyzed by sequence comparisons from *Arabidopsis thaliana* KEA3 and homologous proteins from the monocot *Oryza sativa*, the moss *Physcomitrella patens* and the green alga *Chlamydomonas reinhardtii*. This shows that especially the CPA2 domain is conserved. A percent identity matrix is shown.

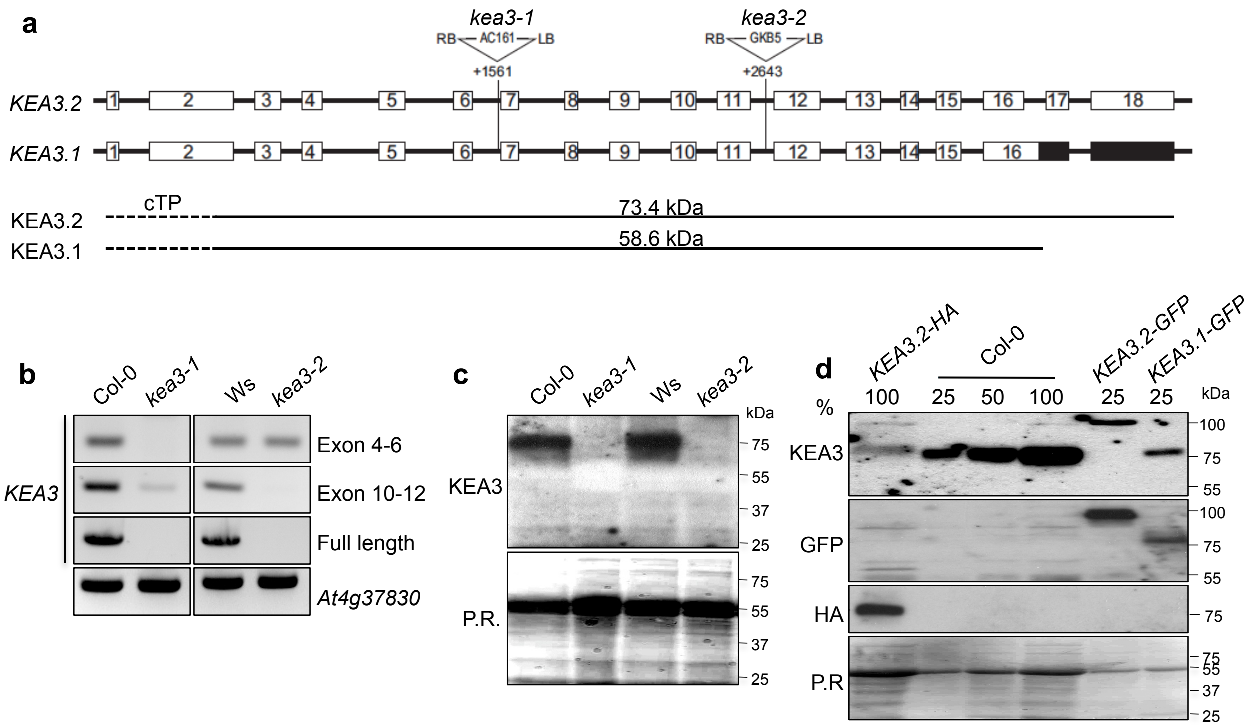

Supplementary Figure 2 | *kea3* mutants lack KEA3, and the major KEA3 isoform is KEA3.2. a, Two different *KEA3* splice forms are annotated in the TAIR database. The translated exons of the two splice forms identified by RT-PCR are depicted as open numbered boxes, un-translated regions as black boxes and introns as connecting black lines. Site and orientation of the T-DNA insertions of the two mutant *kea3* alleles employed in this work are provided. These sites have been confirmed by sequencing of the flanking regions. The sizes of the two mature KEA3 isoforms are shown below. **b,** RT-PCR analysis was performed on total leaf RNA from WT and mutant plants with *KEA3*-specific primers and primers to the unrelated gene *AT4G37830* as positive control using 40 PCR amplification cycles. **c,** Protein blot analysis was performed on total leaf extract from WT and mutant plants using a custom KEA3 antibody derived against the epitope NQLGRKAADFLDERLDPGE present in both isoforms. Ponceau Red (P.R.) was used to stain the membrane to visualize loading and transfer of total leaf protein. Only one KEA3 isoform was detected, which ran at the size of the 75 kDa marker band and was absent in both mutants. **d,** *kea3-1* T1 plants transformed with either *KEA3.1-HA* or *KEA3.2-HA* were selected for resistance to kanamycin and high expression of RFP, a second selection marker encoded by the T-DNA. Twenty independent mutants of each genotype were analyzed for expression of KEA3 and HA protein by Western blot. KEA3 and HA were not detected in plants transformed with *KEA3.1-HA*, suggesting that either the KEA3.1-HA protein or transcript was not stable. The native KEA3 ran only slightly lower than KEA3.2-HA, closer to the expected size of KEA3.2 of 73.4 kDa than to the expected size of KEA3.1 of 58.6 kDa. A KEA3.1-GFP fusion protein ran at similar size as the native KEA3 (the GFP-tag adds 28.2 kDa).

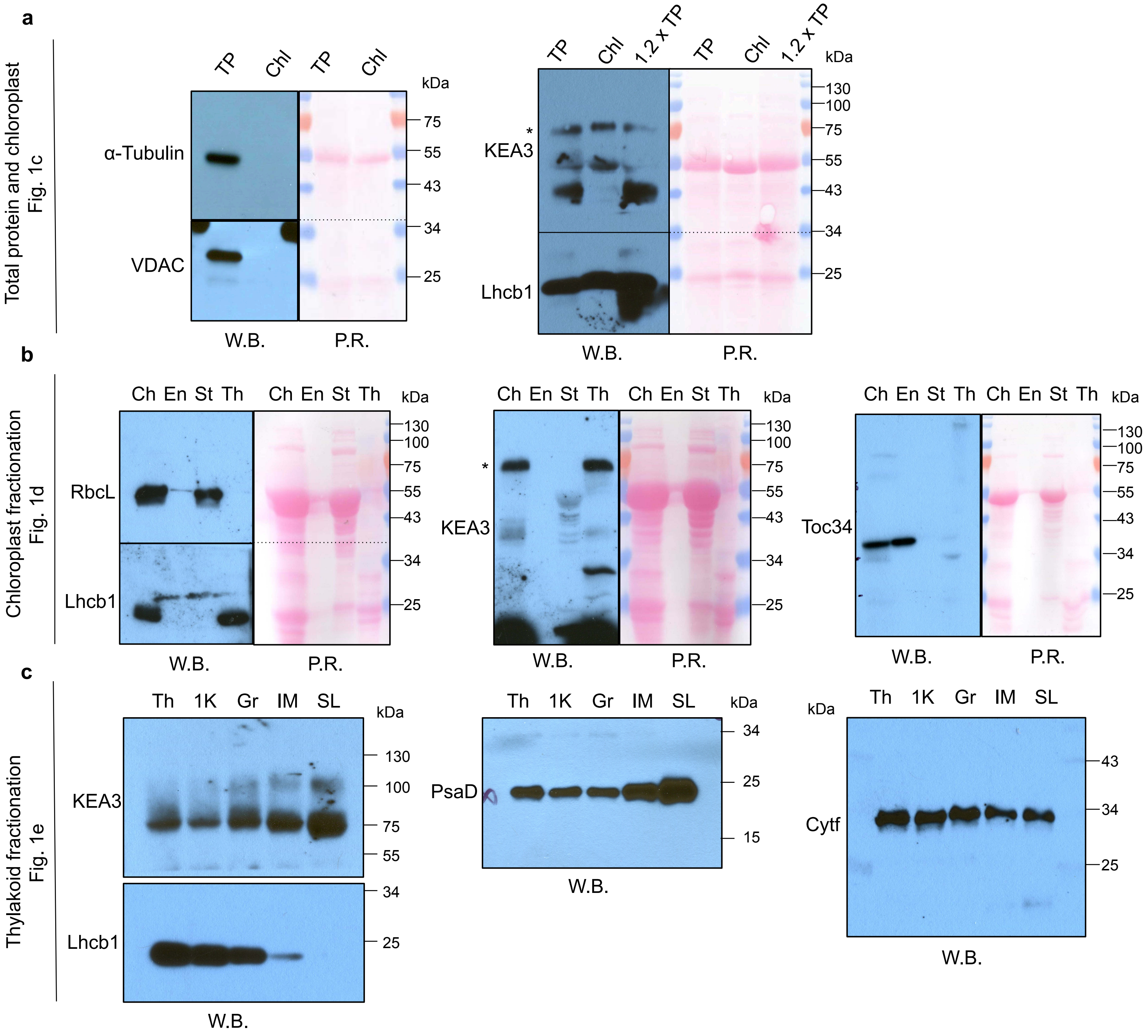

Supplementary Figure 3 | KEA3 localizes to the thylakoid stromal lamellae. a,b,c, Uncropped versions of the westernblots shown in Fig. 1. After transfer of proteins from SDS-PAGEs to nitrocellulose sheets, the membranes were stained with Ponceau red (P.R.) and probed with the indicated antibodies. Signals of the westernblots (W.B.) were detected using ECL and X-ray films. For **a** and **b** corresponding W.B. and P.R. are shown for each blot. Cutting of membranes prior to antibody hybridization is indicated with a dashed line. A representative P.R. for W.B. shown in **c** is presented in Figure 1e. **a, b,** Asterisks (*) indicate the specific KEA3.2 band at 75 kDa.

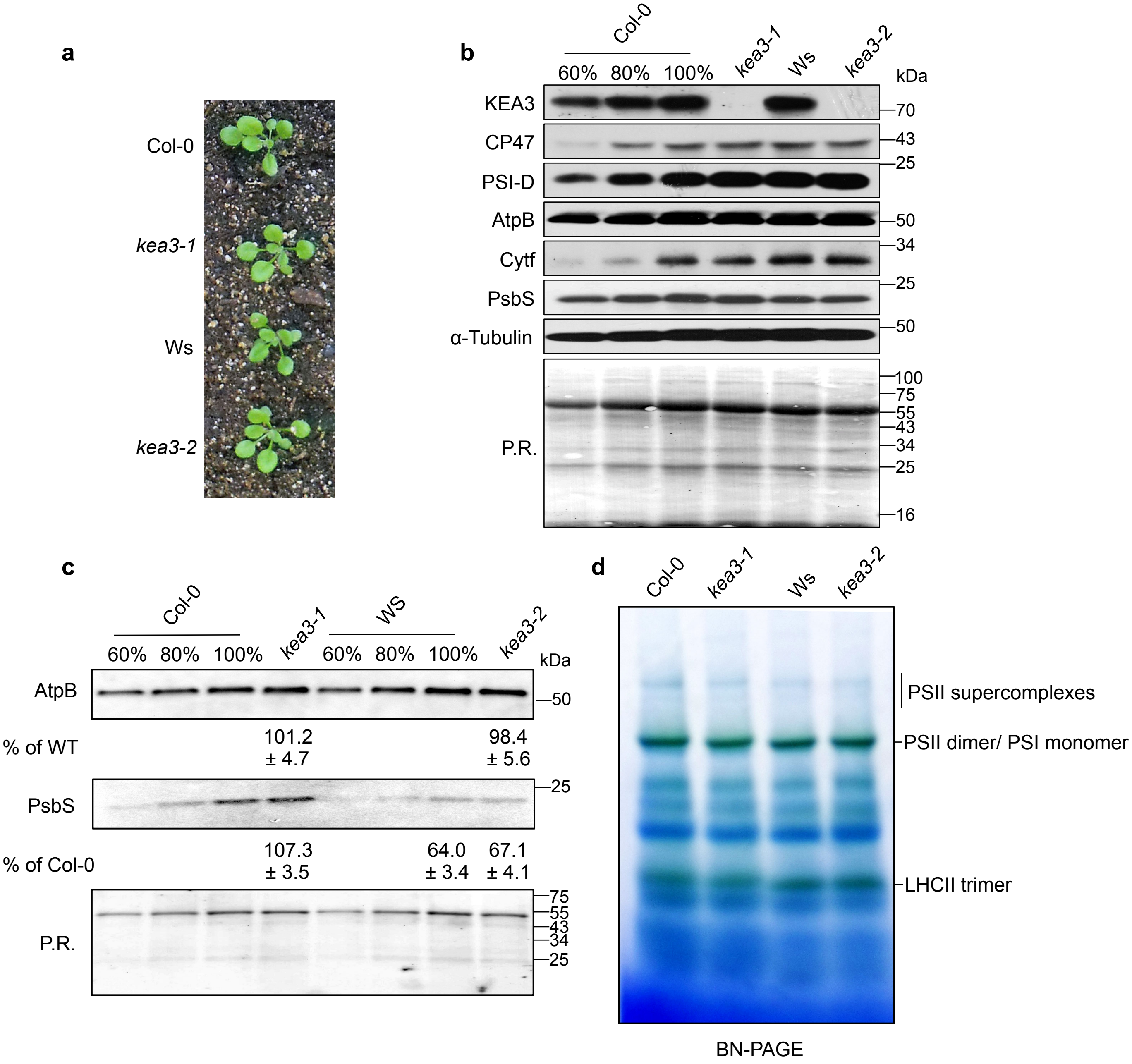

Supplementary Figure 4 | Growth, pigmentation and levels of photosynthetic complexes appear unchanged in *kea3* mutants. a, 17-day old *kea3* mutant (*kea3-1*, *kea3-2*) and corresponding WT (Col-0, Ws) plants were imaged. **b**, Total leaf proteins were separated by SDS-PAGE, transferred to nitrocellulose membranes and probed with antibodies against specific subunits of thylakoid photosynthetic complexes (CP47, PSII; PSI-D, PSI; AtpB, ATP-synthase; Cytf, Cyt b_6f complex) and PsbS. As controls, a ponceau-red (P.R.) stained membrane after protein transfer and a hybridization against the cytoplasmic α-tubulin subunit are shown. **c,** Total leaf extract was prepared as in b and membranes were probed with antibodies against AtpB and PsbS. Relative protein levels in each sample were obtained by normalizing AtpB and PsbS signals to the signal of the P.R stained membrane. A standard curve was calculated from the signals of the WT dilutions and protein levels were calculated according to the equation of the standard curve. Indicated relative protein levels (AtpB % of the corresponding WT; PsbS % of Col-0) correspond to mean values ± s.e.m. of two biological and three technical replicates. **d,** Thylakoids were isolated, complexes were solubilized with 0.7 % n-dodecyl-β-D-maltoside and proteins were separated by Blue-native (BN)-PAGE.

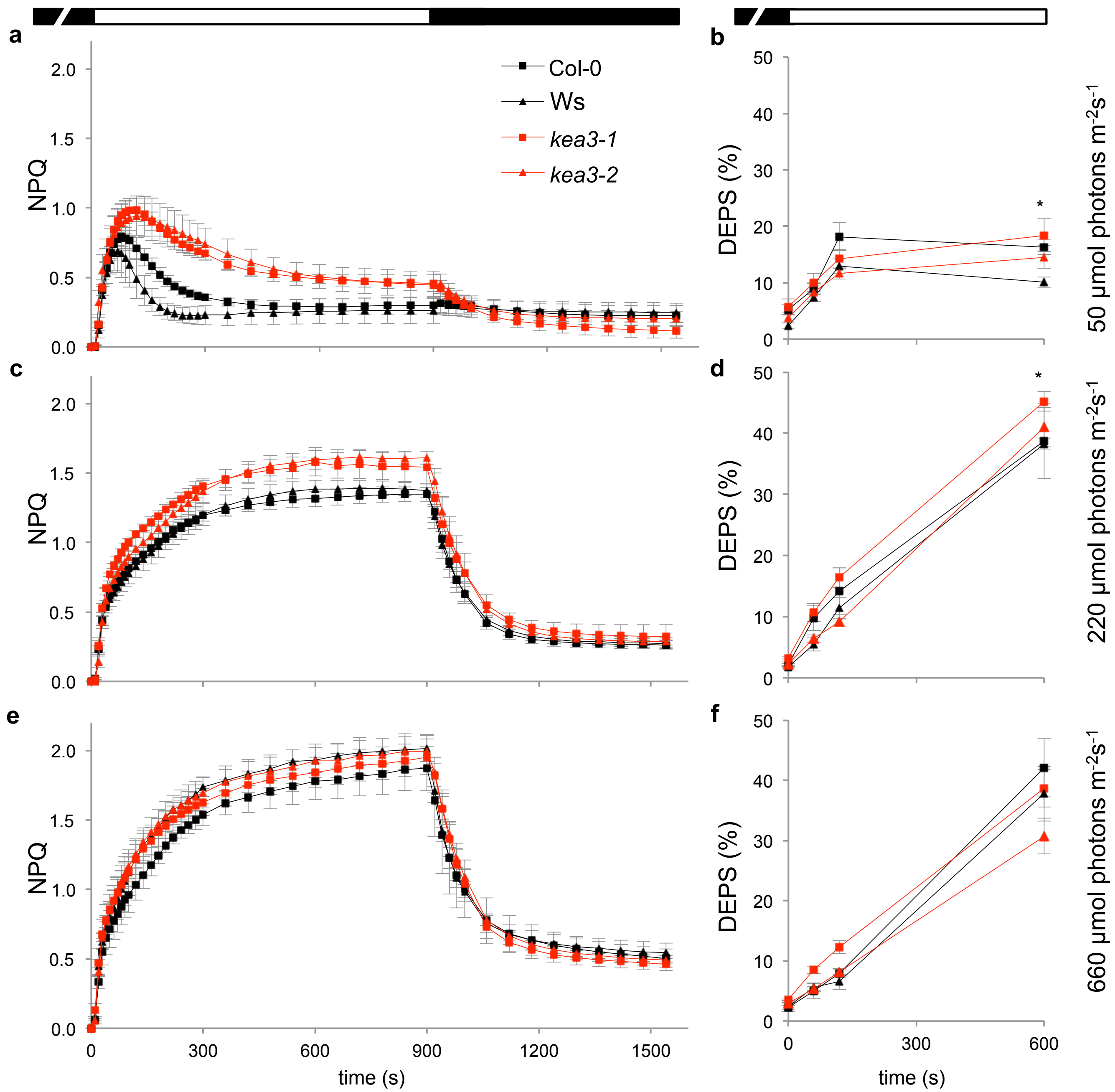

Supplementary Figure 5 | The *kea3* NPQ phenotype is specific for induction at low light intensities and correlates with increased DEPS levels after 10 min. NPQ induction and de-epoxidation state (DEPS, expressed as percentage of the sum of violaxanthin + antheraxanthin + zeaxanthin) were measured by Dual-PAM and HPLC, respectively, in four-week-old wild-type (Col-0, Ws) and mutant (*kea3-1* and *kea3-2*) plants. Leaves were dark-acclimated for 30 min before illumination at light intensities of 50 µmol photons $m^{-2}s^{-1}$ (**a**,**b**), 220 µmol photons $m^{-2}s^{-1}$ (**c**,**d**), and 660 µmol photons $m^{-2}s^{-1}$ (**e**,**f**), for 15 min followed by a 12 min dark relaxation period. Mean values ± s.e.m. of four (NPQ) and 12 (DEPS) independent experiments are shown. During the transition from dark to low light Col-0 shows a higher NPQ response than Ws. This is most likely due differences in the qE machinery and/or in the rate of cyclic electron transport around PSI between the two ecotypes , e.g. PsbS levels are lower in Ws (Supplementary Data Figure 3c). Lower luminal pH in the *kea3* mutants would be expected to increase activation of the violaxanthin de-epoxidase, increasing the de-epoxidation state (DEPS) of the xanthophyll antennae pigments (*24*). Consistent with this, a slight increase ($p<0.05$, ANOVA) in the de-epoxidation state (DEPS) of the xanthophyll pigments was found in *kea3* mutants after 10 min of illumination with 50 and 220 µmol photons $m^{-2}s^{-1}$. (**b**,**d**) Asterisks (*) indicate a difference between both mutants and their corresponding mutants at a given time point with $p<0.05$, Student's *t*-test.

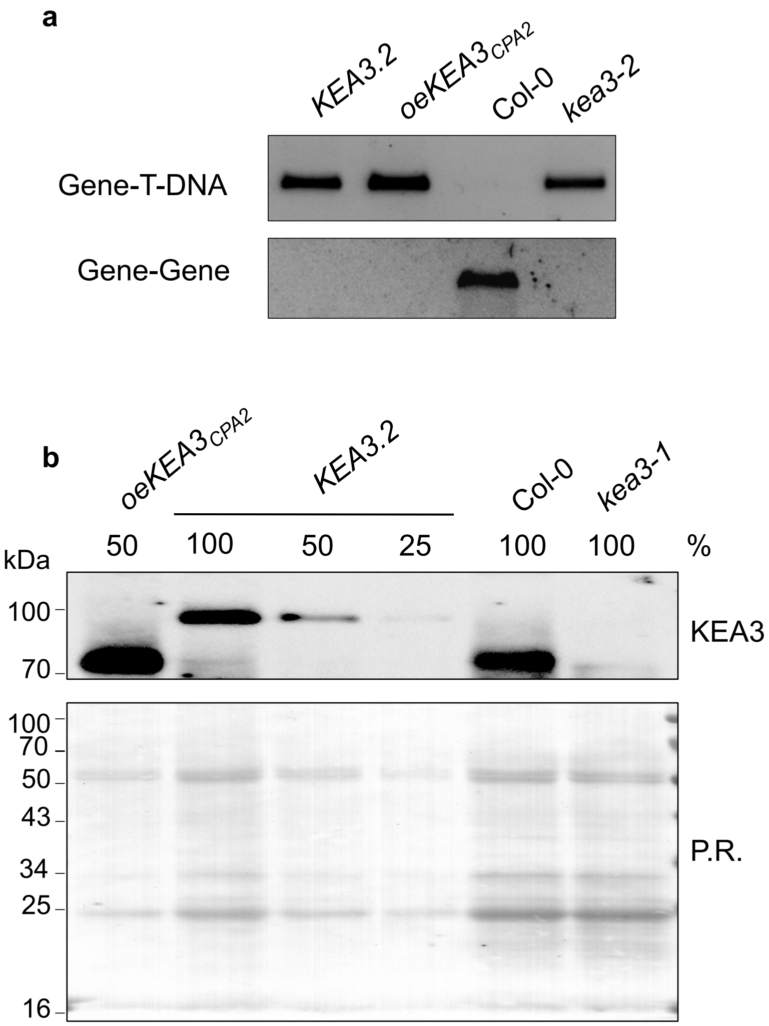

Supplementary Figure 6 | KEA3-GFP is expressed at similar levels to the native KEA3, while $\mathbf{KEA3_{CPA2}}$-GFP is overexpressed. Twenty-five *kea3-1* plants transformed with pB7FWG2 containing the *KEA3.2* sequence were selected for GFP fluorescence and subsequently analyzed for fusion protein levels with the KEA3 antibody. The plant with highest KEA3.2-GFP levels (*KEA3.2*) was used for localization and complementation analysis. Of two *kea3-1* plants transformed with pB7FWG2 containing the $KEA3_{CPA2}$ fragment and selected accordingly, one was expressing the fusion protein ($oeKEA3_{CPA2}$). **a,** DNA from *KEA3.2* and $oeKEA3_{CPA2}$ was analyzed with primers spanning the *kea3* T-DNA insertion site (Gene-Gene) and a T-DNA left border primer with adjacent gene primer (Gene-T-DNA). DNA from Col-0 and *kea3-1* was used as control. **b,** Thylakoid proteins from *KEA3.2*, $oeKEA3_{CPA2}$, Col-0 and *kea3-1* were hybridized with KEA3 antibodies. Ponceau Red (P.R.) stain of the membrane prior to the immunodetection is shown as a second loading and protein transfer control. In *KEA3.2* the fusion protein is present at near-WT levels, and in $oeKEA3_{CPA2}$ it is overexpressed more than two-fold.

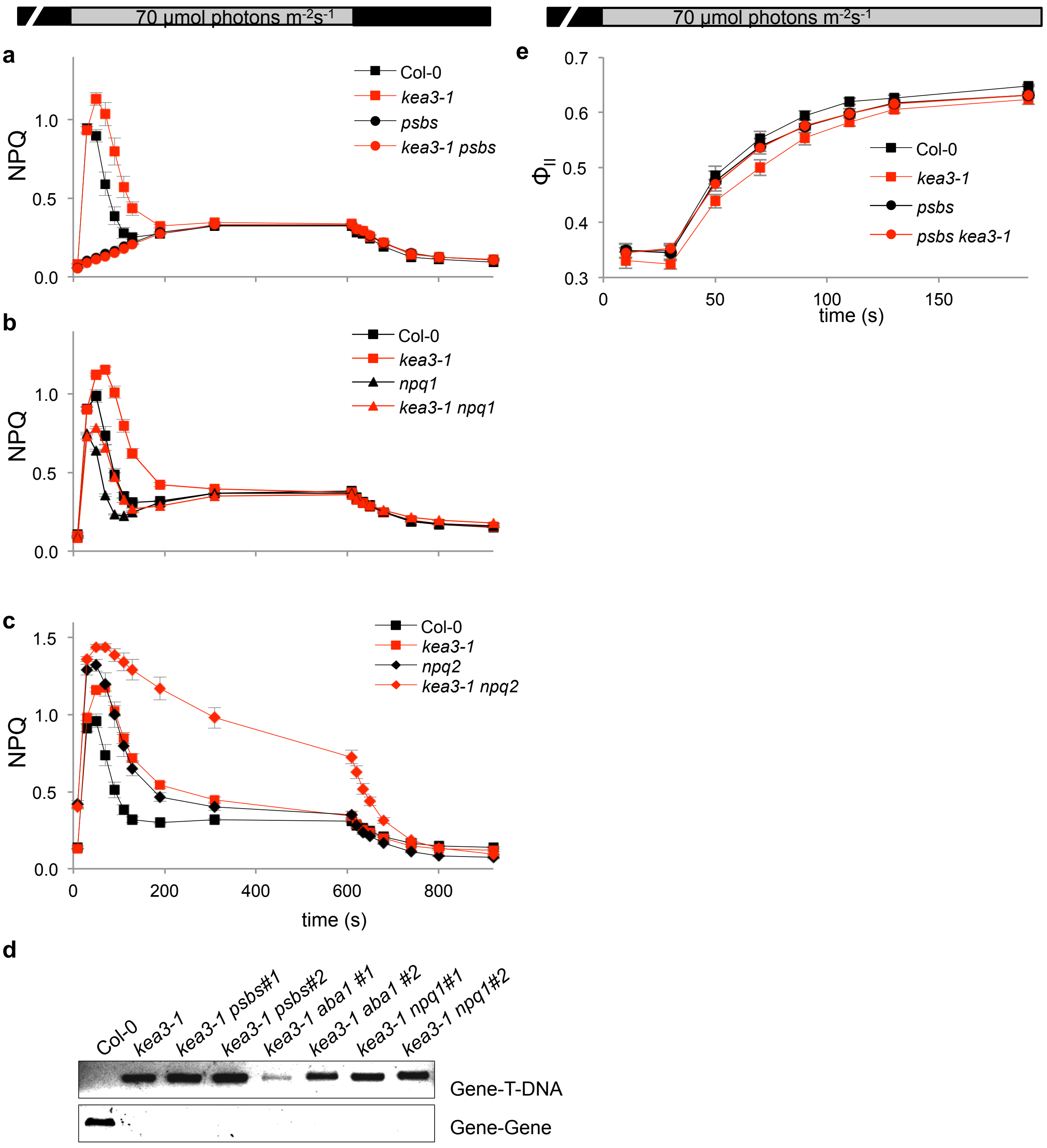

Supplementary Figure 7 | The transient NPQ phenotype in *kea3* is PsbS-dependent and modulated by zeaxanthin; the transient decrease in Φ_{II} observed in *kea3* is PsbS dependent. (**a**,**b**,**c**) NPQ induction in detached leaves of two-week-old plants was measured as in Fig 2A. Error bars represent s.e.m. (n = 6). **d,** Two independent double mutant lines were analyzed for (a,b,c). PCR confirms that all were double mutants with *kea3-1*. **e,** Φ_{II} was calculated from the same experiment as in (a).

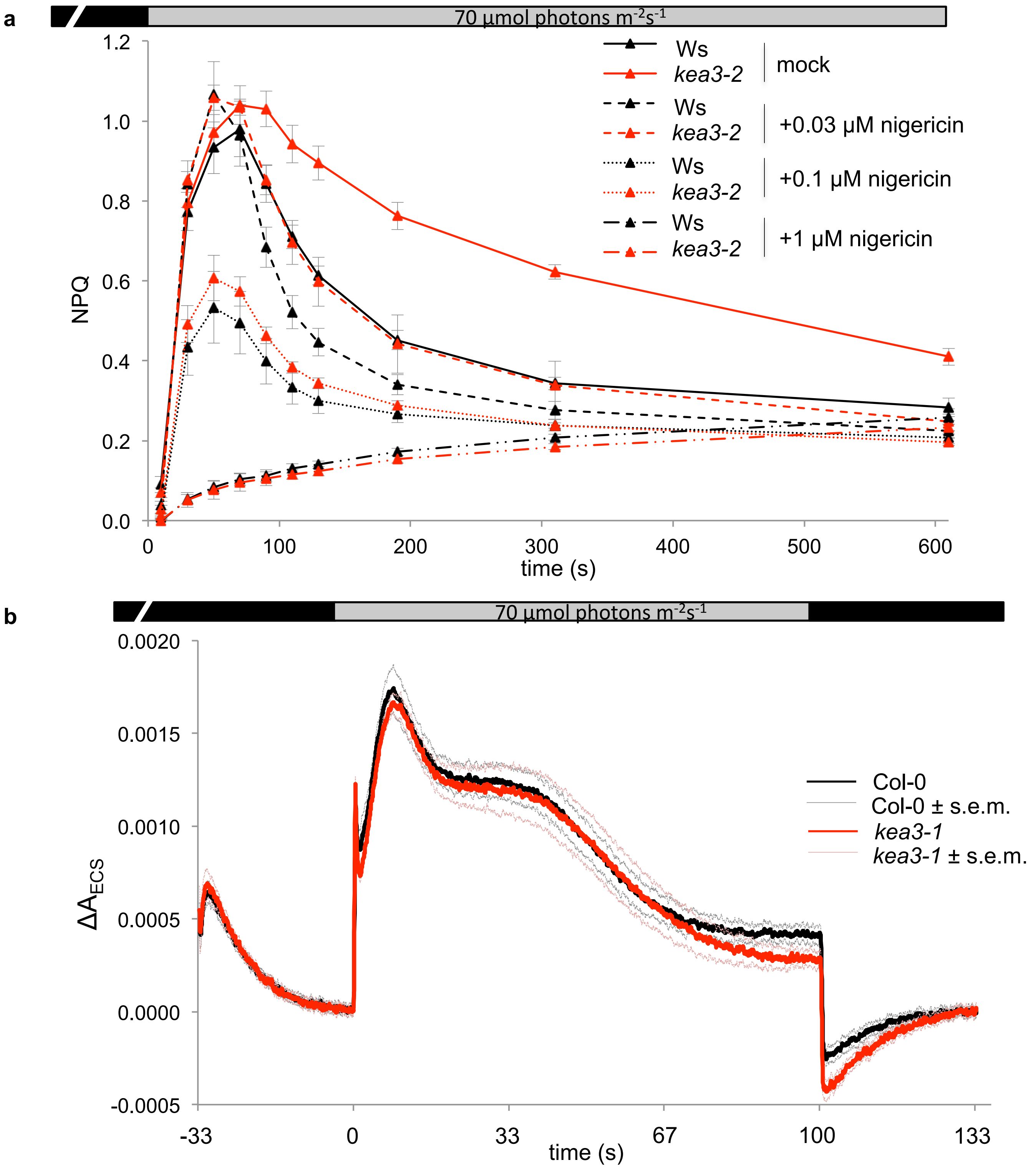

Supplementary Figure 8 | Nigericin rescues the *kea3* NPQ phenotype at low concentrations; and eliminates transient NPQ at high concentrations. After 100 s of low light, *kea3* mutants show increased ΔpH and decreased Δψ. a, NPQ induction upon transition from dark to 70 µmol photons m^{-2} s^{-1} light was measured in WT (Ws) and *kea3-2* leaves incubated in water (mock) or with indicated concentrations of nigericin. Error bars represent s.e.m. (n = 6). **b,** Plants were dark adapted for 30 min, and then illuminated for 100 s with 90 µmol m^{-2} s^{-1}. Then, the light was turned off and full ECS decay kinetics were recorded as absorption change at 520 nm (deconvolved by subtracting a baseline between 505 nm and 535 nm) to measure ΔpH and Δψ in WT (Col-0) and *kea3-1*. The average without and +/- s.e.m. of 6 independent measurements per genotype was plotted as a moving average with interval 5.

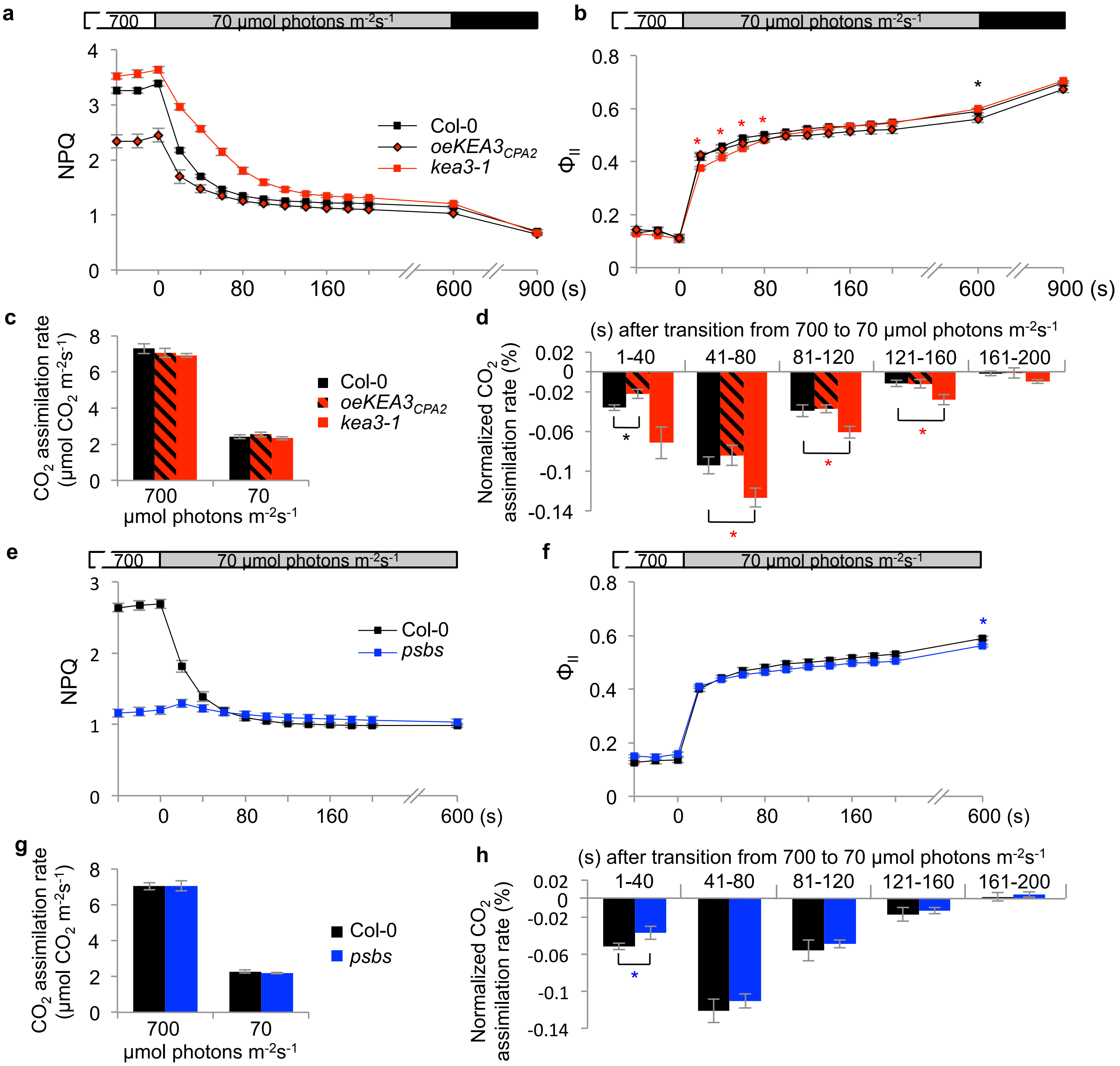

Supplementary Figure 9 | The relative CO2 assimilation rate after a shift from high to low light is increased in *oeKEA3$_{CPA2}$* and *psbs* mutants. **a**, **e**, *oeKEA3$_{CPA2}$* and *psbs* have lower NPQ in high light and upon transition to low light as compared to Col-0. **b**, **f**, Φ_{II} is decreased in low light in *oeKEA3$_{CPA2}$* and *psbs* as compared to Col-0. Φ_{II} was calculated from the same measurement as in (a,e) respectively. **c, g**, *oeKEA3$_{CPA}$* and *psbs* show similar CO_2 assimilation rates during steady state high (700 µmol photons $m^{-2}s^{-1}$) and low light (70 µmol photons $m^{-2}s^{-1}$) as compared to Col-0. **d**, **h**, The normalized CO_2 assimilation rate (as described in Fig. 4) is increased in *oeKEA3$_{CPA2}$* and *psbs* in the first 40 s after the transition to low light, when NPQ is lower compared to Col-0. **a-d**, *kea3-1* behaves very similarly to *kea3-2* upon a transition from high to low light (Fig. 4) with slower NPQ relaxation kinetics, slower recovery of maximum Φ_{II} and a decrease in relative CO2 assimilation as compared to WT (Col-0). **a-h** Error bars represent s.e.m. (n = 6). **b**, **d**, **f**, **h**, Asterisks represent significantly lower Φ_{II} and normalized CO_2 assimilation rate in *oeKEA3$_{CPA2}$* (black), *psbs* (blue), and *kea3-1* (red) as compared to Col-0 ($p < 0.04$).

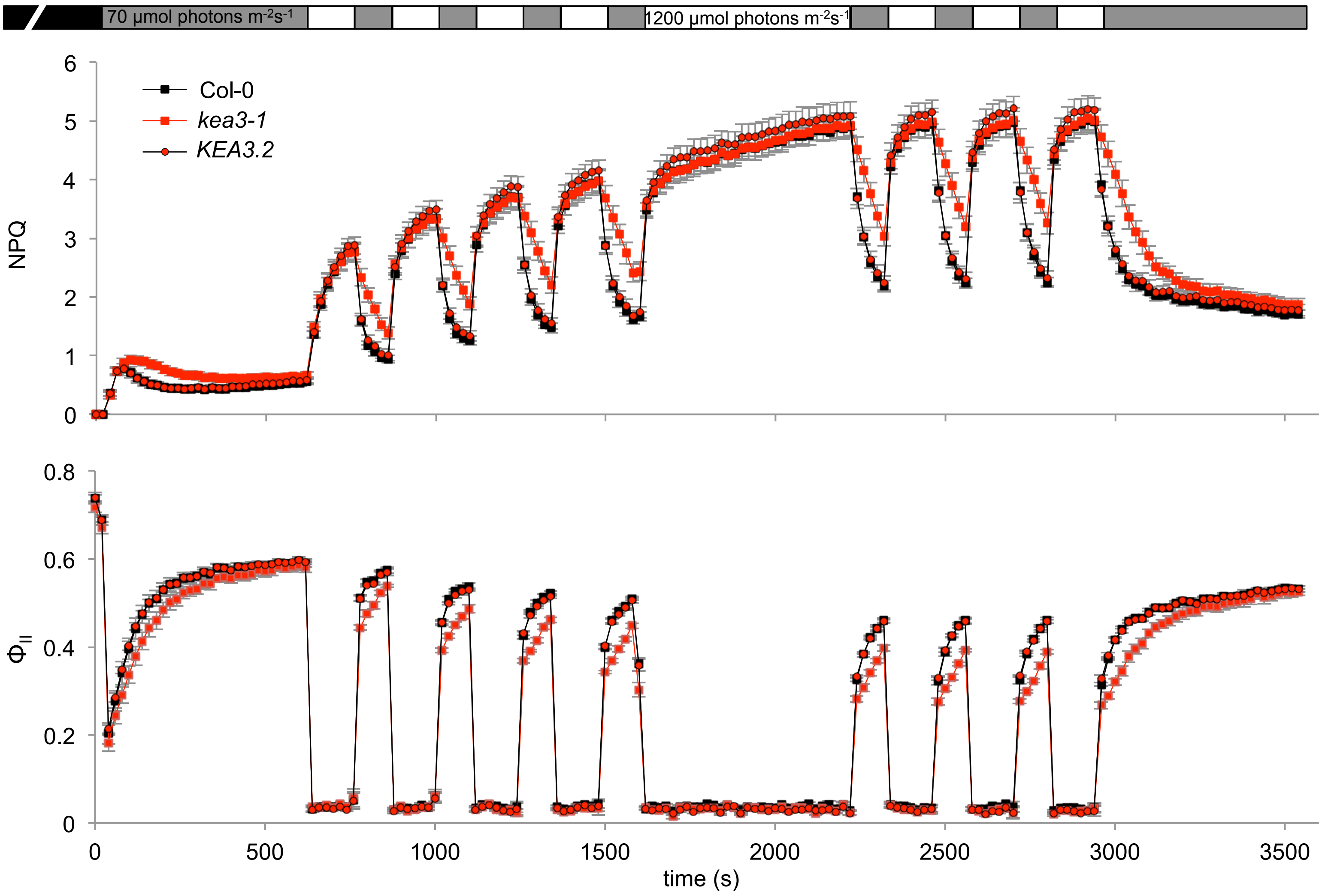

Supplementary Figure 10 | KEA3 regulates the dynamics of photosynthesis in fluctuating light . **a**, In fluctuating light, NPQ relaxation is delayed after each transition from high to low light in *kea3-1*. **g,** Φ_{II} is decreased in *kea3-2* during each transition from high to low light. Φ_{II} was calculated from the same measurement as in **f.** **a**, **b**, Expression of *KEA3.2-GFP* (*KEA3.2*) in *kea3-1* rescues fast NPQ relaxation and recovery of high Φ_{II} . Error bars represent s.e.m. (n = 6).

Gene	Forward primer	Reverse primer	Experiment(s)
KEA3	TAGGGTTTATGGGTGACAACAA	GTCAACTTCAGGAGTTTGCAC	Genotyping *kea3-1*
	AAGCTTTTGTGGCCCTTTGC	TTGGCATGGGACTAACTCAG	Genotyping *kea3-2*<br>RT-PCR exon 10-12
	TTGGCATGGGACTAACTCAG	ACGGCCTCATCAATGCTTCT	RT-PCR exon 4-6
	ATGGCAATTAGTACTATGTTAGG	TTAATCTTGAGCTTTATCAGCTTT	RT-PCR full length
	GGGGACAAGTTTGTACAAAAAAGCAGGCTATGGCAATTAGTACTATGTTAG	GGGGACCACTTTGTACAAGAAAGCTGGGTTATCTTGAGCTTTATCAGCTTTA	Cloning of *KEA3.2* into pDONR for expression in plants
	GGGGACAAGTTTGTACAAAAAAGCAGGCTATGGCAATTAGTACTATGTTAG	GGGGACCACTTTGTACAAGAAAGCTGGGTTCATCTTCACCTCTCCGGGAT	Cloning of $KEA3_{CPA2}$ into pDONR for expression in plants
AT3G07480	CTCTTCAGAAACTCTCCTCTCAA	ATTCCTCTGCGATCTGAACCTC	RT-PCR control

Supplementary Table 1 | Primer combinations used in this work.
